# Regimen of the Japanese Soldier

**Published:** 1888-10

**Authors:** 


REGIMEN OF THE JAPANESE SOLDIER.—On his entrance into the army, the soldier receives a manual of hygiene, in which Ogata has given, in a familiar form, very important counsel. The alimenation of the Japanese soldier is composed of a daily ration of 1,091 grammes of uncooked rice and a money allowance—with which to purchase food—of six cents a day for privates and eight cents a day for officers. With this sum they buy fresh or salted fish; tofou, a pulp of fermented beans, very rich in albumin; daiko or turnip cabbage; onions; radishes; gherkins; batatas, and aquatic plants. They also buy shrimp or lobsters; seeds and stems of nenuphar (*nymphea nelumbo*); preserved ginger; fried or boiled Indian meal; dried mushrooms; preserved red beans; salted plums; fermented cucumbers, etc. To these a small amount of pastry is added, together with allspice, pepper, saffron and fermented sauces, which are used to mask the insipid taste of the rice. The most used of these sauces is shoyou, a fermented product of beans. Rice, which is simply boiled, takes the place of bread. Tea is the only drink, except among those who can afford saki or rice brandy.—*Nouveaux Remedès*.

